# Semaglutide as a potential tool in pre–lung transplant weight loss optimization

**DOI:** 10.1210/jcemcr/luag040

**Published:** 2026-04-01

**Authors:** Roshaneh Ali, Holly Keyt, Carolina Solis-Herrera, Maria Escobar-Vasco

**Affiliations:** Department of Internal Medicine, University of Texas Health Science Center at San Antonio, San Antonio, TX 78229, USA; Division of Pulmonary and Critical Care, University Health Transplant Institute, Alvarez Center for Transplantation, Hepatobiliary Surgery and Innovation, University of Texas Health Science Center at San Antonio, San Antonio, TX 78229, USA; Division of Endocrinology, University of Texas Health Science Center at San Antonio, San Antonio, TX 78229, USA; Division of Endocrinology, University of Texas Health Science Center at San Antonio, San Antonio, TX 78229, USA

**Keywords:** GLP-1 receptor agonist, semaglutide, obesity, lung transplant, COPD

## Abstract

Patients with end-stage lung disease go through an extensive screening process prior to transplant. Obesity and uncontrolled type 2 diabetes mellitus (T2DM) are unfavorable risk factors that lead to poor outcomes. We present the case of a 69-year-old man with stage IV chronic obstructive pulmonary disease (COPD) on chronic oxygen, T2DM on insulin, and class II obesity (reference range, body mass index [BMI], 35.0-39.9) who underwent pre–lung transplant evaluation. He had a BMI of 38.05, surpassing the institutional transplant eligibility criteria of BMI <32. The patient was initiated on semaglutide for weight loss. After 6 months, the patient's BMI decreased to 30.5, losing 25 kg and qualifying him for transplant. However, given substantial improvements in respiratory status, the pre–lung transplant committee deferred waitlisting. After 16 months of treatment, the patient lost a total of 35.17 kg, his forced vital capacity improved from 44% to 82%, and he was weaned off oxygen. Chronic hypoxia and corticosteroids make weight management challenging for COPD patients. This case demonstrates the use of semaglutide for rapid weight loss and improved respiratory function in patients with end-stage lung disease, emphasizing its emerging potential in pre–lung transplant optimization.

## Introduction

Obesity has been established to worsen health outcomes in patients with end-stage lung disease [1]. Typically, obesity is tackled with a holistic approach, involving behavioral, lifestyle, physical, and medication interventions to improve prognosis. Sedentary lifestyle and poor dietary choices contribute to this growing epidemic. Nevertheless, steroids for recurrent exacerbations in advanced chronic obstructive lung disease (COPD) may chronically affect weight and glycemic control. Hypoxia may also limit mobility, making it difficult to exercise. For these patients, obesity rapidly progresses morbidity and mortality in an already end-stage disease.

COPD is one of the leading indications for lung transplantation. Obesity and uncontrolled diabetes are high-risk markers for poorer post–lung transplant outcomes. In a retrospective review by Lederer et al [1], patients with a body mass index (BMI) > 30 had an increased mortality of 22% after transplant, regardless of type of advanced lung disease, disease severity at transplantation, and other potential confounders. In prospective cohort study, obesity was also an independent risk factor for early graft dysfunction, attributed to the chronic inflammatory state seen in obesity [2]. Current guidelines classify class II obesity (reference range, BMI >35-39.9) or greater as being high risk, and transplant is generally not recommended [3]. Thus, early identification and intervention of reversible risk factors can lead to better prognoses.

Various weight loss methods have been trialed for pretransplant optimization. Medications such as orlistat and phentermine-topiramate have been used with variable efficacy [4]. Bariatric surgery is a promising option but is invasive for pretransplant and posttransplant patients [5]. While pulmonary rehabilitation programs improve respiratory function and exercise tolerance, they have not demonstrated significant weight loss [6].

Rapid, noninvasive weight-loss strategies are urgently needed. Glucagon-like peptide-1 receptor agonists (GLP-1RAs), such as semaglutide, have been effective for uncontrolled type 2 diabetes mellitus (T2DM) and obesity in the general population. The SUSTAIN 6 trial also demonstrated that semaglutide reduced cardiovascular major adverse events such as nonfatal stroke, nonfatal myocardial infarction, and cardiovascular death in patients with diabetes, and the SELECT trial thereafter suggested similar cardiovascular benefits in patients with obesity alone [7, 8]. Nevertheless, the effects of semaglutide on lung function have not been specifically evaluated in clinical trials or retrospective reviews. To explore this further, we present a case of a patient with advanced COPD and explore the effects of semaglutide on weight loss and respiratory function.

## Case presentation

A 69-year-old man with a history of COPD, T2DM, and class II obesity **(**BMI of 38.05) presented to the endocrinology clinic for pre–lung transplant optimization. Oxygen saturation ranged from 89% to 92% at rest on 2 to 5 L/min of oxygen (reference range, >88%), and he could walk 135 m on his 6-minute walk test (6MWT) (reference range, >500 m). He had a 40 pack-year smoking history and quit 1 year prior. His pulmonary function tests revealed a forced expiratory volume in 1 second (FEV1) of 26% (reference range, >80%), staging the patient as GOLD 4, group D, or very severe COPD. He was maximized on respiratory therapy using an inhaled corticosteroid, long-acting β-agonist, long-acting muscarinic antagonist, roflumilast, and chronic prednisone 10 mg daily with minimal symptomatic improvement. The patient's BMI, airflow obstruction, dyspnea, and exercise capacity (BODE) score was 8, suggesting a predicted median survival of 3 to 4 years.

## Diagnostic assessment

Barriers to transplantation included obesity, with the institutional criteria requiring a BMI <32. T2DM was reasonably controlled with a glycated hemoglobin A_1c_ of 7.1% (reference range, <7.0%), and the patient required 72 units of insulin glargine daily. Despite also taking liraglutide 1.8 mg daily, he did not have significant insulin dose reduction after 3 months. Liraglutide was discontinued and semaglutide was started at 0.5 mg weekly until a maximum dose of 2 mg weekly was achieved in approximately 8 weeks. He tolerated the medication well and was without symptoms. Subsequent changes in weight, FEV1, 6MWT, and insulin dose were monitored over the course of treatment and are summarized in Table 1.

**Table 1:** Respiratory and metabolic measures over time on maximal semaglutide

	Reference	0 months	6 months	12 months	16 months
BMI	<32 kg/m^2^	38.9 kg/m^2^	30.5 kg/m^2^	29.2 kg/m^2^	27.5 kg/m^2^
Weight		127.3 kg	102 kg	97.5 kg	92.1 kg
Insulin		74 units	36 units	36 units	36 units
FEV1	≥80%	26%	37%	39%	28%*^a^*
FVC	≥80%	44%	70%	78%	87%
FEV1/FVC	≥0.7	0.6	0.5	0.5	0.3
DLCO	80-120%	45%	53%	NA	59%
6MWT distance	>500 m	135 m	256 m	200 m	240 m*^b^*
6MWT frailty score		9	12	12	12
SpO_2_	>88%	95%	94%	94%	95%
Supplemental oxygen		3L	1L	1L	NA
BODE score	<3	8	4	5	5
No. of exacerbations since last visit		NA	2	0	1

Abbreviations: 6MWT, 6-minute walk test; BMI, body mass index; BODE, body mass index, airflow obstruction, dyspnea, and exercise capacity; DLCO, diffusing capacity of the lungs for carbon monoxide; FEV1, forced expiratory volume in 1 second; FVC, forced vital capacity; NA, not available; SpO_2_, oxygen saturation.

^*a*^The patient reported exacerbation during this clinic visit.

^*b*^Mobility limited by recent knee injury.

## Treatment

After 6 months of therapy, including 8 weeks of pulmonary rehabilitation, the patient's BMI decreased to 30.5, losing 25 kg (55 pounds). FEV1 improved from 26% to 37%, and FVC improved from 44% to 70% (reference range, >80%). Concurrently, diffusing capacity of the lungs for carbon monoxide (DLCO) improved from 45% to 53% (reference range, >80%). He could walk 256 m on his 6MWT (reference range, >500 m), required no oxygen at rest, and only 1 L of oxygen with exertion. Furthermore, his insulin requirements decreased from 74 units of glargine to 36 units daily.

## Outcome and follow-up

Although the institutional BMI contraindication to transplant no longer applied, the pre–lung transplant committee deferred active waitlist status given significant respiratory improvements. After 16 months of therapy, the patient's BMI further decreased to 27.01, losing a total of 35.18 kg since starting maximum semaglutide therapy. At 16 months, his FVC improved from 44% to 82% and DLCO improved from 45% to 59%. Of note, his FEV1 decreased from 37% to 28%, likely due to an exacerbation a few weeks prior. After 16 months of treatment, the patient no longer required oxygen with exertion and reported substantial symptomatic improvement. He continues to be followed by the pre–lung transplant evaluation committee in the outpatient setting.

## Discussion

The effects of GLP-1RAs have demonstrated remarkable outcomes both in diabetic and obese populations, and their use has already been explored in the transplant world. Budding research on GLP-1RAs in kidney and liver transplant recipients suggests safety in immunosuppressed patients and efficacy in weight loss, although its use in transplant remains off-label [9-12].

This case highlights semaglutide as a rapid weight-loss agent in patients with advanced lung disease. A growing number of studies have demonstrated the success of semaglutide in pre–lung transplant optimization [13, 14]. In Stryker et al [13], 3 patients with T2DM, class II to III obesity, and end-stage lung disease were started on semaglutide and experienced weight loss and improved diabetes all within at least 6 months of semaglutide use. Interestingly, end-stage lung disease was due to pulmonary arterial hypertension or interstitial lung disease, contrasting with our COPD case. In a retrospective review by Goebel et al [14], 42 patients at a weight-loss clinic with BMI > 32 were pending lung transplant and were grouped by use of GLP-1RA vs alternative weight-loss methods. In the GLP-1RA group, 66.7% (n = 14) achieved their weight goal compared to 52.4% (n = 11) in controls. Importantly, this study did not specify the type of GLP-1RA used, and neither study commented on how respiratory function changed with semaglutide.

In this case, the patient experienced improvement in many respiratory markers. Respiratory improvements are, in part, explained by the restrictive effects of obesity on chest wall compliance. Due to thoracic fat accumulation, chest expansion is decreased. In addition, intra-abdominal pressure increases so downward diaphragmatic movement is limited. Combined, these effects result in reduced resting lung volumes and reduced FVC. With weight loss, increased FVC enhances lung volumes and reduces dyspnea. On the other hand, FEV1, influenced more by COPD than obesity, remains largely unchanged [15].

Beyond lung volumes, this patient also had improved 6MWT distance, reduced oxygen requirements, and improved DLCO. Numerous studies have shown semaglutide reduces systemic inflammatory markers, independent of weight loss [16-18]. In a current phase 3 randomized controlled trial of patients with metabolic dysfunction–associated steatotic liver disease, semaglutide 2.4 mg weekly led to a significant reduction in high-sensitivity C-reactive protein compared to placebo [19]. Thus, respiratory improvements may also be caused by anti-inflammatory effects of semaglutide, underscoring the need for further exploration of its effects on inflammatory modulation in end-stage lung disease.

Currently, semaglutide is not routinely used in patients with end-stage lung disease or in lung transplant due to concerns for aspiration. This followed case reports of pulmonary aspiration during intubation despite following standardized fasting protocols [20-22]. Further research thereafter suggested that while GLP1-RAs may delay gastric emptying, response is variable and that the actual risk of worsening reflux, aspiration, and subsequent aspiration pneumonia has not been identified [23-26]. Thus, multisociety consensus now recommends that patients without risk factors and already on GLP1-RA therapy for some time may continue use in the perioperative setting without reservation [27]. Although lung transplant recipients are inherently at an increased risk of aspiration, the risk of semaglutide-induced aspiration in the nonoperative setting remains unknown [28]. Furthermore, while semaglutide may optimize a patient pre transplant, duration of therapy following lung transplant remains unclear. Patients who lose weight with GLP1-RAs often regain weight following cessation of therapy, suggesting that semaglutide may need to be continued lifelong [29]. Currently, there are no clinical trials evaluating the effects of weight loss, diabetes control, respiratory changes, or risks of aspiration while on semaglutide post transplant.

Limitations include the unclear contribution of comorbidities to respiratory burden. While pulmonary function tests were heavily interpreted in the context of ongoing weight loss, this case cannot establish a causal relationship between semaglutide and respiratory improvement. Without randomized trials, we cannot attribute respiratory improvements to solely weight loss alone. Additionally, the effects of semaglutide on other end-stage lung diseases (ie, interstitial lung disease, cystic fibrosis, bronchiectasis) must also be further investigated.

Nevertheless, our patient was ultimately deferred for transplant because of semaglutide-related clinical improvements. Lung transplant carries its own increased risks of aggressive immunosuppression, recurrent hospitalizations, chronic pneumonias, and acute or chronic lung allograft dysfunction. When compared to other solid organ transplants, lung transplant recipients have among the lowest rates of long-term survival and the highest rates of rehospitalization [30]. Thus, perhaps semaglutide, through improvements in respiratory function, weight, and quality of life, may supersede the need for transplant altogether. This would drastically decrease costs, utilization of health-care systems, organ shortages, and complications from immunosuppression.

The potential of semaglutide as a tool in pre–lung transplant optimization must not be underestimated. Transplant patients are typically treated by interdisciplinary teams, and pretransplant care should be no different. For patients with obesity undergoing transplant evaluation, semaglutide may be considered early as a noninvasive intervention to improve glycemic control, decrease BMI, and alleviate respiratory burden.

## Learning points

- Obese patients with end-stage lung disease are at an increased risk for poor outcomes after transplant.
- Semaglutide can cause rapid weight loss for pre–lung transplant optimization.
- Through weight loss, semaglutide can increase lung volumes and decrease respiratory burden in patients with obesity and lung disease.
- Further investigation on the anti-inflammatory effects of semaglutide in the lungs is needed.

## Data Availability

Data sharing is not applicable to this article as no datasets were generated or analyzed during the current study.

## Abbreviations

6MWT: 6-minute walk test
BMI: body mass index
BODE: body mass index, airflow obstruction, dyspnea, and exercise capacity
COPD: chronic obstructive pulmonary disease
DLCO: diffusing capacity of the lungs for carbon monoxide
FEV1: forced expiratory volume in 1 second
FVC: forced vital capacity
GLP-1RA: glucagon-like peptide-1 receptor agonist
SpO2: oxygen saturation
T2DM: type 2 diabetes mellitus
